# Emerging bioinformatics approaches for analysis of NGS-derived coding and non-coding RNAs in neurodegenerative diseases

**DOI:** 10.3389/fncel.2014.00089

**Published:** 2014-03-27

**Authors:** Alessandro Guffanti, Alon Simchovitz, Hermona Soreq

**Affiliations:** ^1^Laboratory of Molecular Neuroscience, Department of Biological Chemistry, The Edmond and Lily Safra Center of Brain Science, The Hebrew University of Jerusalem Jerusalem, Israel; ^2^Bioinformatics, Genomnia srlMilano, Italy

**Keywords:** neurodegenerative diseases, bioinformatics and computational biology, next-generation sequencing, non-coding RNA, biological networks

## Abstract

Neurodegenerative diseases in general and specifically late-onset Alzheimer’s disease (LOAD) involve a genetically complex and largely obscure ensemble of causative and risk factors accompanied by complex feedback responses. The advent of “high-throughput” transcriptome investigation technologies such as microarray and deep sequencing is increasingly being combined with sophisticated statistical and bioinformatics analysis methods complemented by knowledge-based approaches such as Bayesian Networks or network and graph analyses. Together, such “integrative” studies are beginning to identify co-regulated gene networks linked with biological pathways and potentially modulating disease predisposition, outcome, and progression. Specifically, bioinformatics analyses of integrated microarray and genotyping data in cases and controls reveal changes in gene expression of both protein-coding and small and long regulatory RNAs; highlight relevant quantitative transcriptional differences between LOAD and non-demented control brains and demonstrate reconfiguration of functionally meaningful molecular interaction structures in LOAD. These may be measured as changes in connectivity in “hub nodes” of relevant gene networks (Zhang etal., 2013). We illustrate here the open analytical questions in the transcriptome investigation of neurodegenerative disease studies, proposing “*ad hoc*” strategies for the evaluation of differential gene expression and hints for a simple analysis of the non-coding RNA (ncRNA) part of such datasets. We then survey the emerging role of long ncRNAs (lncRNAs) in the healthy and diseased brain transcriptome and describe the main current methods for computational modeling of gene networks. We propose accessible modular and pathway-oriented methods and guidelines for bioinformatics investigations of whole transcriptome next generation sequencing datasets. We finally present methods and databases for functional interpretations of lncRNAs and propose a simple heuristic approach to visualize and represent physical and functional interactions of the coding and non-coding components of the transcriptome. Integrating in a functional and integrated vision coding and ncRNA analyses is of utmost importance for current and future analyses of neurodegenerative transcriptomes.

## INTRODUCTION

Recently emerging bioinformatics analyses of integrated microarray, next generation sequencing (NGS) and genotyping data in brain and peripheral blood cell samples from neurodegenerative disease cases and matched healthy controls consistently reveal changes in gene expression of both protein-coding and small and long regulatory non-coding RNAs (ncRNAs); highlight relevant quantitative transcriptional differences between demented and non-demented control brains and demonstrate reconfiguration of functionally meaningful molecular interaction structures that may be measured as changes of connectivity in “hub nodes” of relevant gene networks (Karni et al., 2009; Zhang et al., 2013). These developments call for constructing complete and coherent tool kits whereby the contributions of these specific groups of transcripts to the initiation and progression of disease will be elucidated.

“Regulatory” ncRNAs predominantly affect the expression and/or functioning of protein-coding genes. NcRNAs show different biogenesis routes and modes of action, and can be broadly classified based on their size. Small RNA species of less than 200 nucleotides include the microRNAs (miRNAs), which have already emerged as important modulators of development, homeostasis and disease by regulating protein levels, mainly at the post-transcriptional stage. Recent evidences suggest that miRNAs may also directly regulate transcription by interaction with the promoter region of divergently transcribed genes (Matsui et al., 2013), suggesting new insights in the complex relationship between small RNAs, longer transcripts and the quantitative ratio between them.

A substantial fraction of longer transcripts (>200 bp) in mammalian genomes do not code for proteins and are usually expressed at a low level. These are classified according to their relative position with respect to the coding gene structure and include long ncRNAs (lncRNAs) and long intergenic ncRNAs (lincRNAs). LncRNAs (also known as processed transcripts) by definition are found within protein-coding genes, overlapping with promoters, exons or introns in either sense or antisense orientations. LincRNAs, on the other hand, are always found in intergenic regions. There is increasing evidence that lncRNAs are involved in brain development and that different lncRNAs are expressed in different neuroanatomic areas, and possibly acting on chromatin; hinting at a regulatory function at the spatio-temporal level of gene expression (Mercer et al., 2008). It is now widely accepted that lncRNAs can have numerous molecular functions, including modulating transcriptional patterns, regulating protein activities, serving structural or organizational roles, altering RNA processing events, and serving as precursors to small RNAs.

The number of lncRNA species increases in genomes of developmentally complex organisms, which highlights the importance of RNA-based levels of control in the evolution of multicellular organisms (Fatica and Bozzoni, 2013). Most common neurodegenerative diseases of the human brain, however, are either not detected in other species, or else they manifest themselves in different ways. Furthermore, a significant fraction of ncRNAs are primate-specific. Taken together, these two pieces of evidence may suggest that the progressive disruption of regulatory lncRNAs plays an important role in neurodegenerative syndromes. However, current systems-level analyses of gene regulatory networks are primarily focused on protein-coding genes, which make up a mere 2% of the human transcriptional output but whose cellular functions are better understood. That highly structured ncRNAs interact with chromatin or provide docking sites for binding proteins or other RNAs suggests that they bridge the gap between protein complexes and sequence information encoded in the genome. This hidden layer of RNA regulatory networks may be central to developmental and homeostatic processes, and its deregulation could be consequently involved in degenerative neurological disorders such as Alzheimer’s and Parkinson’s disease.

The different and diverse modes of action of regulatory RNAs adds another level of complication, in that such regulation would not necessarily change the observed level of expression of the tested coding transcripts but may block or support their functioning in other upstream or downstream ways. This implies that the customary use of threshold-dependent technologies may miss part of these effects and mask others, and calls for developing threshold-independent analysis modes. Based on all of these considerations, we propose here the concepts and tools for functional investigation of full-transcriptome next-generation sequencing datasets, with focus on both the coding and non-coding ensembles. We examine in a first instance the statistical aspects linked with the evaluation of differential expression in these systems. We then analyze the relevance of ncRNAs in neurodegeneration. We suggest a strategy to implement a network-based integrated exploration of the outcome of differential gene expression analysis. We introduce lncRNAs Finally, we suggest a strategy to integrate and display the coding and ncRNA aspects in a gene network. Such a bioinformatics procedure could be then adapted and applied to original experimental data in late-onset Alzheimer’s disease (LOAD) and other neurodegenerative diseases, where complex cell diversities are involved and no drastic transcriptional changes are measured between disease and control as opposed, for instance, to cancer studies.

## GUIDELINES FOR TRANSCRIPTOME DIFFERENTIAL EXPRESSION ANALYSIS APPLIED TO NEURODEGENERATIVE DISEASES

Systematic transcriptome study in neurodegenerative diseases such as amyotrophic lateral sclerosis, Parkinson’s, and Alzheimer’s diseases (AD) has advanced considerably in recent years, alluding to common patterns such as dys-regulation of genes related with neuroinflammation, splicing, intracellular signaling pathways and mitochondrial dysfunctions (Cooper-Knock et al., 2012). In LOAD, the best distinction refers to cognitive deterioration; hence, cognitive stratification of samples may help to identify gradual transcriptome changes along disease progression.

Our laboratory (Barbash and Soreq, 2012) recently proposed a novel strategy to explore brain transcriptome datasets from cognitively stratified patients at different disease stages for unifying the AD molecular patterns involved in disease initiation and progression. Conventional threshold-dependent analysis methods identify transcripts that are drastically modified in AD, ignoring those within-threshold transcripts whose level was only marginally changed. However, if each member of a group of genes relevant to AD etiology is marginally up-regulated, one might expect a relevant pathological state in the observed tissue even in the absence of major gene changes. In this threshold-independent approach, therefore, we compared the distribution of changes in a well-defined gene group with the global distribution of the experiment. This method allows identification of cumulative changes in groups of genes defined by a common parameter: acting in the same pathway, located in the same cellular organelle, and so on. This approach has been applied to the meta analysis of a large number of microarray datasets (Barbash and Soreq, 2012), contributing to the identification of coherent and progressive early onset hippocampal-specific changes in biological processes such as synaptic transmission, protein folding and RNA splicing known to be affected in end stage AD, but for which the dynamics was not yet reported in the literature.

Starting from this background, we reasoned that many relevant gene changes in AD and other neurodegenerative diseases may go unnoticed also in the differential expression analysis of whole transcriptome NGS datasets, which in addition to known exons and transcripts identifies previously unknown regulatory RNAs. There are two main conceptual starting points for the analysis of this kind of data. The older, and widely used, strategy (TopHat/Cufflinks/Cuffdiff) starts from sequence assembly and transcript reconstruction, performs abundance estimation and evaluates differential expression (Trapnell et al., 2010). Cufflinks constructs a parsimonious set of transcripts that “explain” the reads observed in a RNA-seq experiment, doing so by reducing the comparative assembly problem to a mathematical problem (maximum matching in bipartite graphs). It works particularly well with paired reads; and in systems where there is a relevant change in gene expression associated with a relative amount of change in alternative splicing. Nevertheless, apparent changes in neurodegenerative disease transcriptomes may reflect relevant and massive changes in the alternative splicing pattern, while being accompanied by complex modest changes in gene expression (Berson et al., 2012). Therefore, it is doubtful that this algorithm can handle well the kind of analyses that are associated, for instance, with cognitive stratification of LOAD samples.

A second and more recent strategy for the analysis of NGS transcriptome datasets is based on the assumption that the correct distribution for modeling the distribution of reads on the target genome is a binomial negative, and that an “*ad hoc*” normalization method should be employed (Robinson et al., 2010). This method; however does not (yet) provide relevant information on the structural transcript variations, since it is based on read counts associated with each gene, and hence it is insensitive to at least part of the relevant splicing changes associated with the progression of neurodegenerative events. On the other hand, for the same reason, statistical values [false discovery rate (FDR)] will be often reported as non-significant due to the small and widely distributed changes in gene expression that may only be detected by the non-threshold methods. A third analytical strategy consists of using a method very similar to the primary analysis of microarray datasets: perform upper quantile normalization of the values of gene-associated Read Counts per Million (i.e., the read count scaled to 1 million for each sample); evaluate differential expression with an exact *t*-test; and correct multiple testing using the Benjamini–Hochberg approach.

**FIGURE 1:**
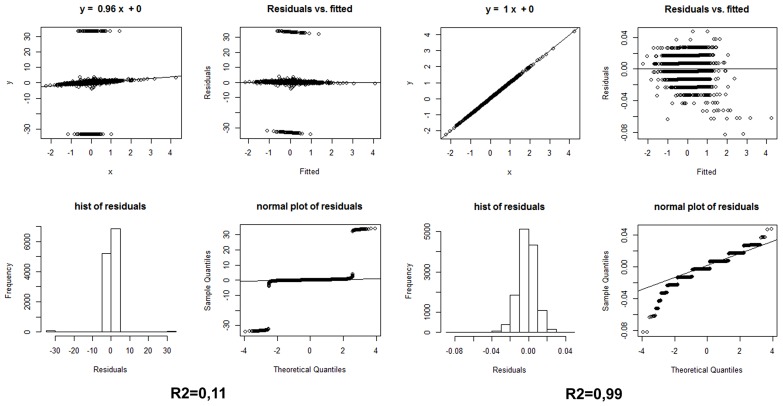
(Left) Statistics on the differential expression results of AD whole transcriptome samples vs. non-demented controls, calculated by edgeR and Cufflink/Cuffdiff, sorted, and compared by Log2 Fold Change only, i.e., without any FDR threshold. (Right) Same statistics on the same samples, using the* Log2 Fold Changes *elaborated with edgeR and the simple Fisher test procedure described in the text. Hist: histogram.

A strategy we propose here for the evaluation of ranked differential gene expression in neurodegenerative diseases, especially in cohorts stratified by cognitive deterioration, is to apply to the same samples two different differential gene expression methods from the three which we have listed above; correlate by sign and compare the Log2 Fold Change values, without in a first instance imposing a statistical threshold or even considering the FDR values; finally, to apply a simple linear model with residual plots to evaluate the statistics and the residuals. Only those genes that show the same sign of variation between two methods, with a Log2FC of at least ±0.40, should be included in a differentially expressed gene list for the subsequent functional analyses. The statistic (corrected *P*-values) of the method that worked better for the FDR evaluation should confirm the generation of reliable results with this simple strategy. **Figure 1** shows the effect of comparing the same samples (AD vs. non-demented healthy controls) using two different methods: Cufflinks and edgeR vs. Cufflinks and Fisher. The different convergence of methods will produce non-correlated, or correlated, lists of differentially expressed genes with the same sign, when sorted by Fold Change. Examining **Figure 1**, it is readily apparent from the correlation plots and values and from the residuals histogram and normal plot that only analyzing these NGS whole transcriptome datasets with the intersection of edgeR and a method based on upper quantile normalization and Fisher test we will obtain a robust set of differentially expressed genes, upregulated and downregulated. Recent advances in Bayesian methods applied to the analysis of differential expression (Glaus et al., 2012; Bi and Davuluri, 2013) reveal interesting advantages in comparison to the other established methods based on transcript reconstruction or binomial negative read mapping distribution, so they may represent interesting alternatives to the strategy reported here.

Concerning lncRNA transcriptome analyses, important consideration must be given to the choice of reference transcriptome database. A comprehensive specialized lincRNA database has recently become available (Xie et al., 2014; Noncode^1^), and the Ensembl project^2^ and related annotation from the BioMart project/Havana group at the Sanger Institute provide effective identification, classification and counting of differentially expressed non-coding transcriptomes associated with Parkinson’s disease treatment including an elaborate lincRNA subset (Goedert and Spillantini, 2006). Software capable of local alignments such as the latest version of bowtie2^3^ is suggested for ncRNA searches, given the high sequence heterogeneity of these transcripts. Preliminary results of in-house unpublished bioinformatics analyses of whole transcriptomes from early and advanced AD and Control samples identified around 9.900 lncRNA transcripts, of which 600 were differentially expressed in the diseased brains (*P* value < 0.05 for each comparison). Among these annotated ncRNA transcripts we found antisense RNAs; small nucleolar RNA host genes; transcribed pseudogenes and non-coding transcripts from human leukocyte antigen regions. This example highlights a varied non-canonical transcript panorama, which calls for further functional and integrated transcriptome annotation and functional predictions. Section 5 will introduce some updated approaches to tackle this problem.

## THE ROLE OF LONG ncRNAs IN NEURODEGENERATIVE DISEASES

A growing body of evidence links various ncRNAs with neurodegenerative diseases in general and specifically with AD. Both small and long non-coding RNAs are identified as possible suspects in this context by involvement with various neurodegeneration-related processes and proteins; these ncRNAs might be involved both in disease etiology and its progression. Therefore, intensified research of these ncRNAs can assist in both unveiling the mysteries that still remain in the processes underlying various neurodegenerative conditions and in identifying possible candidate target genes for therapeutic interference.

The first example we will describe is that of β-Secretase-1 (BACE1). Cleavage of amyloid precursor protein (APP) to yield the amyloid beta (Aβ) peptide by BACE1 rather than by α-Secretase at a later stage enables the protein’s cleavage by γ-Secretase, which causes the formation of characteristic LOAD neuropathology of aggregated amyloid “plaques” (Faghihi et al., 2008). BACE1 mRNA is regulated by both short and lncRNAs. The transcript is subjected to down-regulation by several miRNAs, as well as to up-regulation by the lncRNA BACE1-anti-sense (BACE1-AS; Faghihi et al., 2008). The miRNA mechanism of operation was already described; but BACE1-AS displays a different mechanism of action. Specifically, the non-coding transcript is partially complementary to the coding transcript. The two bind to form a partially double-stranded RNA, increasing BACE1 mRNA stability – and therefore increasing both BACE1 mRNA and protein levels in the cell (Faghihi et al., 2008). Interestingly, both BACE1 and BACE1-AS transcripts are up-regulated in Alzheimer’s brains, compared to control brains (Faghihi et al., 2008). Therefore, disruption of the regulatory nature of both short and long transcripts might change BACE1 protein levels, either promoting or interrupting with Aβ aggregates formation and consequently with AD pathology.

Sortilin-related receptor-1 (SORL1) is yet another protein that is connected to AD. This protein’s function is still not entirely understood; however, it is believed to be involved in endocytosis, and also in APP recycling. SNPs in this gene have been associated with LOAD, by a possible mechanism of improper recycling of APP that allows the latter’s compartmentalization with BACE1, resulting in Aβ formation (Rogaeva et al., 2007). SORL1 was found to be down-regulated in cerebrospinal fluids removed from AD patients (Ma et al., 2009) – However, an ncRNA transcribed in an antisense fashion from intron1 in the SORL1 gene is up-regulated in post-mortem AD brains. This ncRNA, annotated A51, promotes alternative splicing of SORL1, to the formation of a protein with poorer performance in APP localization, elevating Aβ accumulation and aggregation – hence possibly escalating neurodegenerative events and pushing toward the development of AD (Ciarlo et al., 2013).

Another antisense lncRNA which up-regulates protein levels is ubiquitin carboxyl-terminal esterase L1-AS (UCHL1-AS; Carrieri et al., 2012). UCHL1 is a De-Ubiquitinase highly abundant in the brain. A mutation in this gene was identified in a rare form of a familial Parkinson’s disease (Leroy et al., 1998), known as PARK5 (Lesage and Brice, 2009). A different mutation was found in a rare progressive neurodegenerative disease (Bilguvar et al., 2013). Both mutations lead to the creation of a loss of function protein (Leroy et al., 1998; Bilguvar et al., 2013), indicating that proper de-ubiquitination and UCHL1 amounts are critical to avoid neurodegenerative deterioration. The UCHL1-AS ncRNA binds in its 5′ Region to the coding transcript’s 5′ Region and causes up-regulation of protein levels without affecting mRNA levels (Carrieri et al., 2012). Such change in protein levels might have functional implications, and might have an impact on neurodegenerative pathology without any change in transcript levels.

Another possible aspect of lncRNA involvement in neurodegenerative disease may be in their functioning as competing endogenous RNAs (ceRNA). CeRNAs are transcripts that include miRNA recognition elements (MREs), and are therefore competing with other miRNA targets on miRNA binding, providing a layer of regulation over miRNA function (which is, by itself, regulatory). CeRNAs can be either pseudogenes (Poliseno et al., 2010) or lncRNAs (Cesana et al., 2011). Many lncRNAs with possible MREs have been identified through bioinformatics analyses as being differentially expressed in several neurodegenerative diseases – Huntington’s disease (HD), AD and PD (Costa et al., 2012; Soreq et al., 2014). These lncRNAs might each affect multiple miRNAs, and through them many mRNA targets and the expression of many proteins – possibly explaining at least part of the vast transcriptional differences caused by neurodegenerative disease.

A possible emerging therapeutic aspect for ncRNAs can be observed in HD. HD is a severe progressive neurodegenerative disorder, with a known genetic cause – additional CAG nucleotides repeats in the Huntingtin (htt) gene. The disease involves depletion of brain-derived neurotrophic factor (BDNF) in the caudate and putamen nuclei of the striatum, involved in HD pathology (Ferrer et al., 2000). BDNF is weakly transcribed in the striatum, but is rather efficiently transcribed in the cerebral cortex from where it is anterogradely transported to the striatum (Altar et al., 1997). Over-expression of BDNF in the frontal cortex of HD-model mice seems to improve many of the HD symptoms (Gharami et al., 2008). The lncRNA BDNF-AS, also known as BDNF opposing strand (BDNFOS) is an antisense non-coding transcript to BDNF that down-regulates the amount of BDNF (Modarresi et al., 2012). Inhibition of this antisense transcript by small interfering RNA (siRNA) causes up-regulation of BDNF, both *in vitro* (in mouse and human cell lines) and *in vivo* (in mice; Modarresi et al., 2012). Is it possible to use such siRNA to up-regulate BDNF and ameliorate HD symptoms? Time will tell.

## GENE NETWORKS AND THEIR APPLICATION IN INTERPRETING WHOLE TRANSCRIPTOME DATASETS: AN APPLICATION TO A NEURODEGENERATION STUDY

The cell is an integrated device made of several thousand types of interacting proteins, each of which is a molecular machine that carries out a specific task with precision. Cells live in a dynamical environment, where different situations require different proteins. For instance, when a cell senses a nutrient, or a risk of damage, it reacts accordingly by synthesizing transport channels or repair proteins. The cell therefore continuously monitors its environment and keeps calculating the amounts at which each type of protein is required. This information-processing function largely determines the rate of production and turnover of each protein, and is primarily carried out by gene networks.

The most familiar gene networks illustrate the dynamics behavior of the cell following exposure to an external signal (input): transcription networks where nodes are genes and edges represent transcriptional regulation of one gene by the protein product of another gene. Other gene networks include signaling pathways; functional interaction (FI) networks and modules; physical interaction networks; biochemical networks and so on. Biologically significant gene networks show a set of features which distinguishes them from random networks: the median number of gene connections must be greater than two; the degree distribution of the gene-to-gene connections must exhibit a tail indicating that many genes are poorly connected while few are highly connected (“gene hubs”); gene-to-gene interconnections must indicate that the network is enriched in “cliques,” that is, sets of genes that are all pairwise connected. Such properties of non-random gene-to-gene connections and the structure of these interconnections to form cliques are characteristic of many biological networks (Khanin and Wit, 2006).

Differential equations currently form the most prominent approach for the modeling, analysis and simulation of molecular interaction networks. There is, however, a growing interest in qualitative network analysis approaches, capable of inferring qualitative properties of the system dynamics from the currently available incomplete and non-quantitative data. Non-linear dynamics networks, Boolean networks and graphs are the most popular approaches to qualitative networks, and the last approach is the one mostly used in the applications we present here.

The representation of a gene network (such as a gene regulatory network) as a graph allows the analysis of its structural properties by means of graph-theoretical techniques. The global connectivity properties of the network can, for instance, be described by the average degree and the degree of distribution of the vertices. The degree *k* of a vertex indicates the number of edges to which it is connected; together with the average *k* degree and the *k* degree distribution of the graph, it forms a set of properties that give an indication of the complexity of the graph and allow different types of graphs, and therefore networks, to be distinguished.

A very interesting, comprehensive and recent work compared microarray gene expression datasets between LOAD (376 samples) and control (173 samples) non-demented subjects, using a complex integrated approach (Zhang et al., 2013). Many hundreds of carefully selected brain tissues were profiled both by gene expression analyses using microarrays and by genomic DNA genotype analyses. Gene expression traits showing individual variability in transcript profiles were identified; the correlation (connectivity) strength between differentially expressed genes was calculated, and hierarchical cluster analysis was performed to construct the undirected gene co-expression network. Simultaneously, single nucleotide polymorphisms in brain DNA (eSNPs) were used as causal anchors in the construction of directed relationships among nodes in the network. Comparison of networks in LOAD and non-demented brains was performed to explore any effect on molecular interaction structures associated with the disease. Differentially connected modules in LOAD were investigated for their functional organization, module relevance to clinical outcome, as well as the enrichment of brain eSNPs. Finally, modules were rank-ordered for their strength of the functional enrichment, module correlation to neuropathology, and eSNP enrichment.

While this huge effort could not have detected lncRNAs which may be absent from the employed microarrays, the results highlight interesting functional modules. A module correlated with multiple LOAD clinical covariates was identified as being enriched with immune functions and pathways related to microglia activity. This module includes many classified as members of the complement cascade, such as toll-like receptor signaling, chemokines/cytokines, the Major Histocompatibility Complex, and the Fc-receptor system. This and many other studies on the neuro-inflammation correlates of LOAD supports the notion that targeting genes “located” in the center of the most inter-connected hubs may effectively disrupt disease-related networks for the purpose of therapy.

Another recent computational approach to identify functional network modules possibly implied in AD (Mayburd and Baranova, 2013) starts from gene lists, processed into different tiers of evidence consistently established by enrichment analysis across subsets of the same experiments and across different experiments and platforms. The “Cut-offs” were established through ontological and semantic enrichment, and the resulted shortened gene lists were re-expanded by Ingenuity Pathway Assistant tool^4^. The resulting sub-networks provided the basis for generating mechanistic hypotheses on the AD etiology that were partially validated by literature searches; these were called Compact Disease Model (CDM).

A simple and accessible, yet quite powerful, system for performing functional network analysis starting from gene sets is based on functional protein interaction networks (Wu et al., 2010). The focus here is on biological pathways. Pathway-based hypothesis generation is the basis for several popular data analysis systems, including GOMiner (Zeeberg et al., 2005), Gene Set Enrichment Analysis (Subramanian et al., 2005) and commercial tools such as Ingenuity Systems.

Reactome (Matthews et al., 2009)^5^ is an expert-curated, highly reliable knowledgebase of human biological pathways. Pathways in Reactome are described as a series of molecular events that transform one or more input physical entities into one or more output entities in catalyzed or regulated ways by other entities. Entities include small molecules, proteins, complexes, post-translationally modified proteins, and nucleic acid sequences. Each physical entity is assigned a unique accession number and associated with a stable online database. This connects curated data in Reactome with online repositories of genome-scale data such as UniProt and EntrezGenes; ad makes it possible to un-ambiguously associate a position on the genome with a component of the pathway.

In contrast to pathway databases, collections of pairwise relationships among protein and genes offer much higher coverage but can draw in their results a noticeable number of “false” relationships between gene products, since a physical interaction does not obligatorily include a biological relationship. A FI network (Wu et al., 2010) combines curated interactions from Reactome and other pathway databases with un-curated pairwise relationships obtained from physical protein–protein interactions (PPi’s) networks in human and model organisms, gene co-expression data, protein domain-domain interactions, protein interactions generated from text mining, and gene ontology (GO) annotations. This approach uses a naive Bayes classifier (NBC) to distinguish high-likelihood FIs from non-functional pairwise relationships as well as outright false positive ones.

Cytoscape (Saito et al., 2012)^6^ is an open source software platform for visualizing molecular interaction networks and biological pathways and integrating these networks with annotations, gene expression profiles and other datasets. This software integrates analytical components through the concepts of “plugins,” and a plugin is available for the generation of Reactome FI networks from gene lists^7^.

**FIGURE 2:**
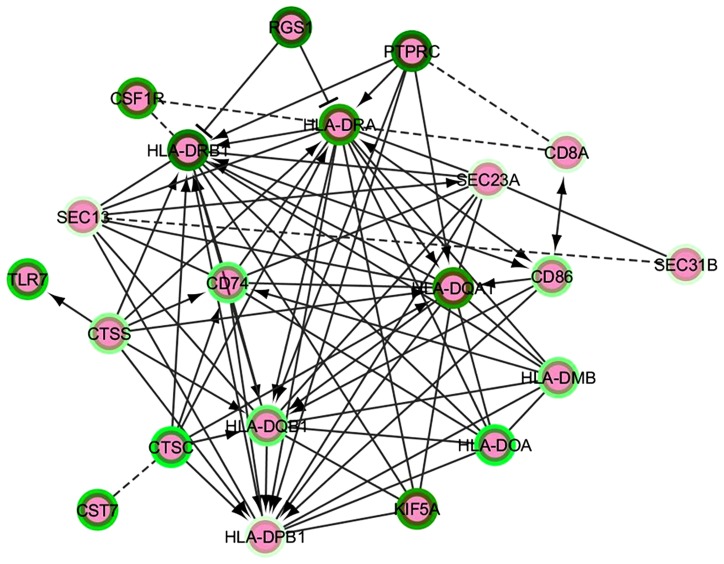
** A functional interaction network module from an AD vs. control NGS dataset analysis**. The intensity of the green color in the border of the nodes represent the Log2FC – darker green corresponds to minor negative Log2FC, i.e., the most down-regulated genes. The edges represent functional interactions detected between the nodes; these can be non-directed, directed stimulatory (arrows), directed repressor (T-shaped edges) or inferred (discontinuous line edges).

Based on all of the above, we propose a strategy to extract functionally connected modules from lists of differentially expressed coding genes by following one of the analytical approaches detailed under “Differential Expression Analysis.” Our strategy involves application of the Reactome search for FI to selected lists of genes with their relative Log2 Fold Change values (up- or down-) in the neurodegenerative brain. Manual selection from Cytoscape of a particular FI network containing a subset of the input gene lists enables to plugin a request to globally evaluate pathway and GO (MF/BP/CC) enrichment within this network. Given the importance and relevance of transcript modules, their FI plugin identification in the main networks of Cytoscape or Reactome should be followed by repeated enrichment analysis on each module. An update to the Reactome FI interaction module has been recently added (in 2013); however, using a more regularly maintained annotation resource such as Ingenuity could be important in terms of the sensibility and specificity of network identification. Enriched pathway lists can then be compared and intersected between different comparisons and conditions, yielding a high-profile view of the main functional clusters mobilized under disease progression or, simply, in the healthy/diseased transition. **Figure 2** presents an example of such a functional module based on NGS differential expression analysis from LOAD compared to non-demented brain and generated from the main FI network. This module highlights a calculated enrichment in down-regulated major histocompatibility complex genes, supporting the recent report by Zhang et al. (2013).

## INTERPRETING LONG ncRNAs THROUGH FUNCTIONAL ANNOTATION AND TRANSCRIPTIONAL NETWORKS; PRIMARY APPLICATION TO A NEURODEGENERATION STUDY

The ncRNA-oriented interpretation of NGS transcriptome datasets in disease (and often in neurodegeneration) studies involves innovative and promising emerging directions. These include integration of the information derived from the differential analysis of coding and ncRNAs based on biomedical annotations reported in specialized resources such as ncRNA databases, Pubmed and OMIM. In addition, interesting semi-supervised learning methods have recently been proposed for efficient classification of lncRNA and disease state based on lncRNA expression profiles and associated comparisons (Chen and Yan, 2013; Yang et al., 2014). A recently proposed global prediction method for functional annotation of lncRNAs (Guo et al., 2013) involves the construction of a so-called “bi-colored” biological network combining two type vertices (protein-coding and non-coding genes) as well as two type edges (co-expression and PPi’s) in the network. A number of case studies, including brain-expressed lncRNAs with predicted neuronal functions, suggest an advantage of bi-colored networks as compared to, for instance, co-expression networks (Guo et al., 2013). Although this approach has only been applied to murine data and is not yet available as usable software, it is clearly a very promising and powerful predictive annotation method.

The rapidly growing list of ncRNA sequences annotated in public databases such as Noncode (Xie et al., 2014)^8^ has reached the noticeable total number of 210,831 lncRNAs in its latest release (v.4). However, immediately usable and well-established methods for understanding the functional role of lncRNAs are still lacking, even for those associated with the phenotype of interest. Likewise, it is still impossible to readily integrate the regulatory roles of differentially expressed lncRNAs with the gene networks of differentially regulated coding RNAs identified in the samples of interest. A simple strategy we propose to address this issue is to integrate the representation of regulatory relationships between coding and non-coding genes in terms of networks by combining the use of publicly available information resources (specialized ncRNA databases, PubMed etc.) and the Cytoscape software. The starting point is a table of regulatory links, e.g., literature search for ncRNAs/coding gene or PPi relationships in neurodegenerative diseases, starting from NGS transcriptome-based ncRNA gene list. **Table 1** presents an example for such a study based on datasets derived from AD vs. normal control brains.

**Table 1:** A selection of known coding/non-coding or coding/coding gene or protein–protein interactions involved in neurodegenerative diseases, derived from the current literature.

Gene_ID	ncRNA ?	TGT_ID	ncRNA ?	Interaction_type	Direction	Pos/Neg	Reference
A51	Y	SORL1	N	Alternative_splicing	>	?	Ma et al. (2009)
BACE1-AS	Y	BACE1	N	Post-transcriptionally_activate	>	+	Faghihi et al. (2008)
17A	Y	GABA B2	N	Alternative_splicing	>	?	Massone et al. (2011)
NMD29	Y	APP	N	Promotes_amyloid beta	>	+	Massone et al. (2012)
HAR1F	Y	REST	N	Repress_transcription	<	-	Johnson et al. (2010)
HAR1R	Y	REST	N	Repress_transcription	<	-	Johnson et al. (2010)
BDNFOS	Y	BDNF	N	Post-transcriptionally_repress	>	-	Ferrer et al. (2000)
UCHL1-AS	Y	UCHL1	N	Post-transcriptionally_activate	>	+	Carrieri et al. (2012)
HTTAS	Y	HTT	N	Post-transcriptionally_repress	>	-	Chung et al. (2011)
BACE1	N	APP	N	Promotes_amyloid beta	>	?	Goedert and Spillantini (2006)
HTT	N	BDNF	N	Promotes_transport	>	+	Gauthier et al. (2004)
UCHL1	N	APP	N	Unknown	?	?	Cottrell et al. (2005)
HTT	N	REST	N	Promotes_proper_localization	>	+	Zuccato et al. (2003)

*The first and third columns report the source and target genes for a given interaction. The second and fourth column report whether the gene or the target is a non-coding RNA. The fifth column reports a range of classified interaction types which have been created “ad hoc” from what is available from the literature. The sixth column reports the direction of the interaction, the seventh column whether this is a positive or a negative interaction, and the last column reports the relevant reference.*

**FIGURE 3:**
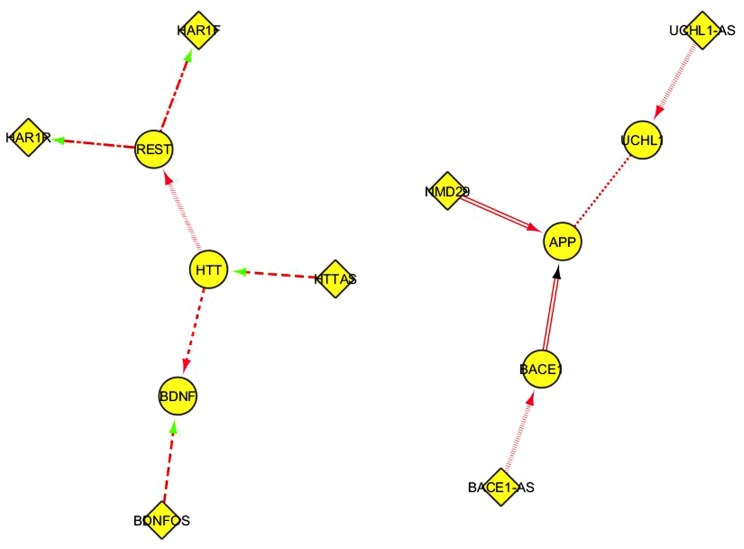
**A Cytoscape network representation of ncRNA/coding RNA and coding RNA/coding RNA manually annotated functional interactions described in Table 1**. Diamonds represent ncRNA genes, ellipses represent coding genes. The functional meaning of the graphical format of the edges, including the color, is explained in the main text.

This table can be suitably reformatted and elaborated in Cytoscape, generating one or more network representation. In **Figure 3**, every interaction type described in Table 1 corresponds to a different edge line format; for instance, the “alternative splicing” relationship is represented by continuous line edges between the nodes; “repress_transcription” by a discontinuous point and segment line; “promotes_amyloid beta” by a double line edge and so on. Diamonds and ellipses represent ncRNA and coding genes, respectably. Directions of edges represent the direction of the FI, with arrow colors specifying positive (red), negative (green), or unknown (black) interactions. Such visual representation of literature-derived lncRNA/coding RNA interactions can be a useful starting point for integrating other functional information associated with the coding genes in these nodes.

A parallel strategy has recently been extended by another group (Xie et al., 2014), generating two distinct networks which can be integrated through a single bipartite lncRNA-disease network: a lncRNA-implicated disease network (lncDN), in which the nodes are disease and the links are lncRNAs; and a disease-associated lncRNA network (DlncN), in which the ncRNAs are nodes and the diseases are edges. Formal network analysis techniques, such as analysis of the network degree of distribution or topology and comparison with random networks highlighted the biological plausibility of this initial representation. The starting approach was the same as the one proposed here [data mining of specialized databases such as LncRNADisease (Chen et al., 2013) and manual paper scanning], and two tables were released: one linking diseases (including AD) with lncRNAs and another linking lncRNAs with other elements such as other RNAs, proteins, transcription factors and so on. These tables could be used to originate representations in Cytoscape with the method described above including also original information from the transcriptome NGS experiment under examination.

Current strategies for constructing databases of interaction networks primarily rely on the “heuristic” interactions derived from careful database and literature scans. Yet more recently, a growing number of databases of RNA–RNA and protein-RNA interactions from CLIP-Seq experiments report annotated interaction networks between miRNA–circRNAs, miRNA–mRNA, and miRNA–lncRNAs (Li et al., 2014; Yuan et al., 2014). In particular, NPinter^9^ is focused on interactions between ncRNAs (excluding tRNAs and rRNAs) and other biomolecules (proteins, RNAs and genomic DNA). These interactions are represented with the same Cytoscape web layout we propose here, are annotated as far as this is currently possible and their use may complement the procedure described above, providing a functional view of entire NGS transcriptome analyses, with focus on lncRNAs.

## Conflict of Interest Statement

The authors declare that the research was conducted in the absence of any commercial or financial relationships that could be construed as a potential conflict of interest.

## Footnotes

1 http://www.noncode.org/

2 http://www.ensembl.org/info/genome/genebuild/ncrna.html

3 http://bowtie-bio.sourceforge.net/bowtie2/index.shtml

4 http://www.ingenuity.com/products/ipa

5 http://www.reactome.org/

6 http://www.cytoscape.org

7 http://wiki.reactome.org/index.php/Reactome_FI_Cytoscape_Plugin

8 http://www.noncode.org

9 http://www.bioinfo.org/NPInter
